# Supplementary material for: Microbial source tracking of human and animal fecal contamination in Ecuadorian households
Source: Appl Environ Microbiol. Author manuscript; Available in PMC 2026 Feb 27. (PMC12838389; doi:10.1128/aem.01694-25)
Supplement: Supplementary Material [file NIHMS2139546-supplement-Supplementary_Material.docx]

Supplementary Material

Microbial source tracking of human and animal fecal contamination in Ecuadorian households

Kelsey J Jesser^*1^, Viviana Alban^*1,3^, Aldo E. Lobos^2^, Javier Gallard-Góngora^2^, Gabriel Trueba^3^, Gwenyth O Lee^4^, Joseph NS Eisenberg^5^, Valerie J Harwood^2^, Karen Levy^1^

*co-first authors

^1^University of Washington, Department of Environmental and Occupational Health Sciences

^2^University of South Florida, Department of Integrative Biology

^3^Universidad San Francisco de Quito, Colegio de Ciencias Biológicas y Ambientales, Instituto de Microbiología

^4^ Rutgers University, Rutgers Global Health Institute

^5^University of Michigan, Department of Epidemiology

**Figure S1:** MST marker sensitivity and specificity (A) and marker abundance in target and non-target samples (B).


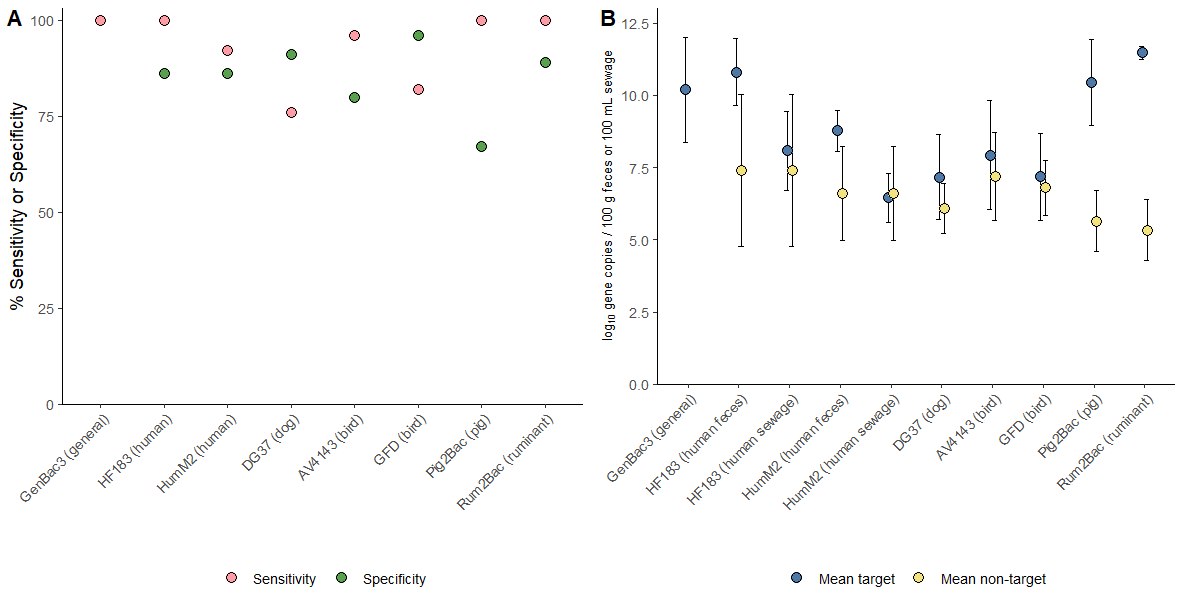


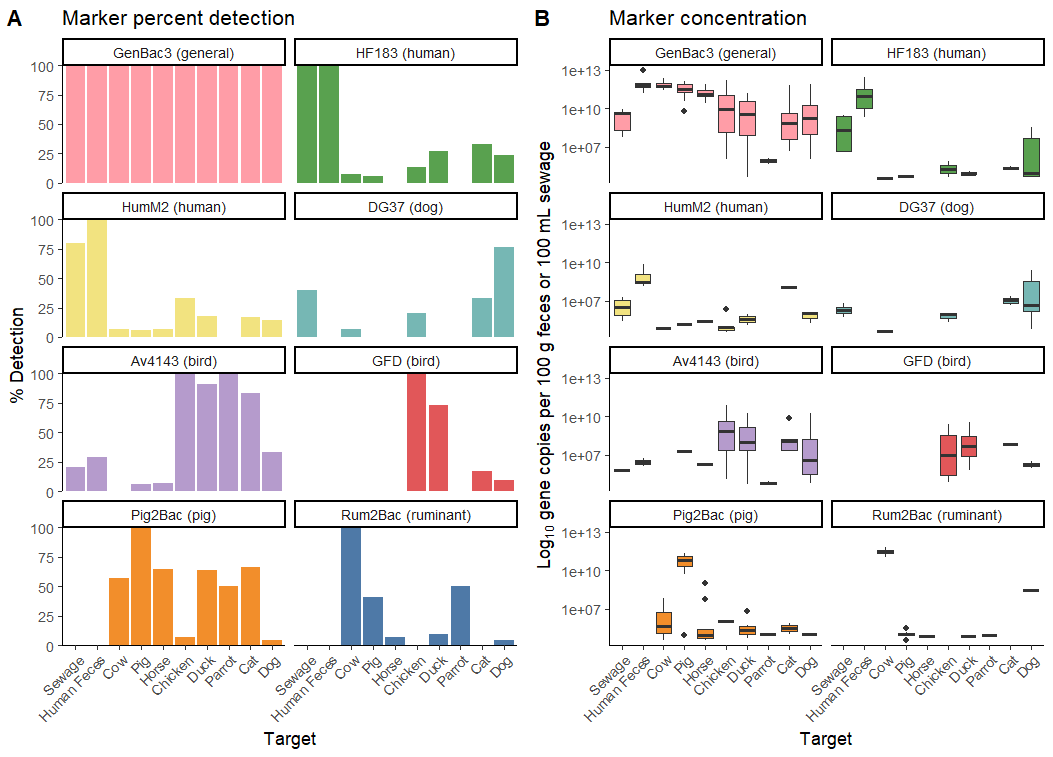


**Figure S2:** MST marker percent detection (A) and concentration (B) in sewage and human and animal feces. Text in parentheses indicates the target host for each candidate MST assay.


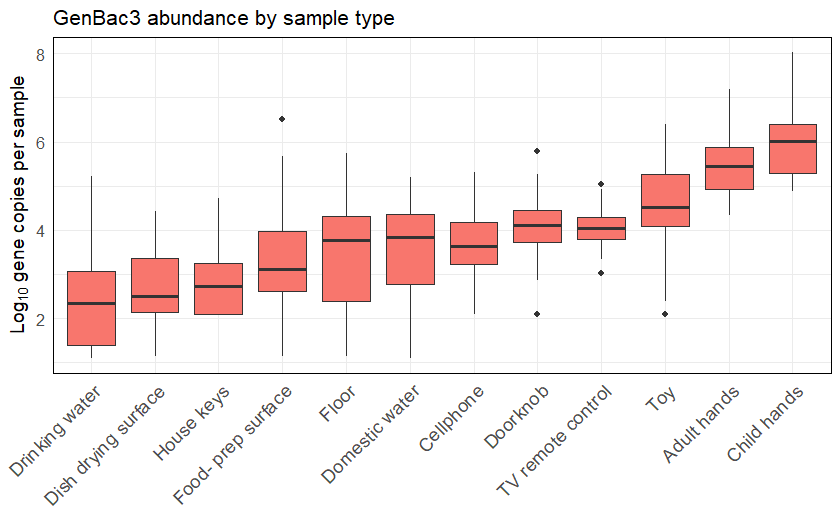


**Figure S3:** Box plot showing GenBac3 MST marker concentrations across various household sample types. Data are presented as log gene copies per pair of hands (child and adult hand rinses), 100 cm^2^ surface area (floors, dish drying and food-prep surfaces), or object (keys, toys, cell phones, TV remotes). Food and soil were excluded from this analysis because there was low prevalence of GenBac3 (<50%) detected in these sample types. Household sample qPCR data were calculated as log_10_ gene copies per 100 g (food and soil), gene copies per pair of hands (child and adult hand rinses), gene copies per 100 cm^2^ surface area (floors, dish drying and food preparation surfaces), and gene copies per object (keys, toys, cell phones, TV remotes)*.*

| **Table S1:** Animal ownership in study households. | | | | | | | | | | | | | |  |
| --- | --- | --- | --- | --- | --- | --- | --- | --- | --- | --- | --- | --- | --- | --- |
|  |  | **Any animal** | **Chickens** | **Ducks** | **Turkeys** | **Guinea pig** | **Dog** | **Pig** | **Cattle** | **Cats** | **Creole**  **chickens** | **Bushrat** | **Other** |  |
| **Number of households reporting animal ownership^1^** | **All study households** | 59 | 9 | 8 | 1 | 1 | 33 | 14 | 1 | 30 | 21 | 0 | 6 |  |
|  | **Urban households** | 15 | 2 | 2 | 0 | 0 | 9 | 2 | 0 | 6 | 3 | 0 | 1 |  |
|  | **Semi-rural household** | 23 | 4 | 2 | 1 | 1 | 12 | 6 | 0 | 14 | 7 | 0 | 3 |  |
|  | **Rural household** | 21 | 3 | 4 | 0 | 0 | 12 | 6 | 1 | 10 | 11 | 0 | 2 |  |
| **Average animals owned per household (when present)** | **All study households** | 5.7 | 13.6 | 5.1 | 2.0 | 3.0 | 2.0 | 2.7 | 8.0 | 1.5 | 15.8 | na | 1.7 |  |
|  | **Urban households** | 3.9 | 10.5 | 3.5 | na | na | 1.3 | 3.5 | na | 1.5 | 2.7 | na | 3.0 |  |
|  | **Semi-rural household** | 6.3 | 8.5 | 4.5 | 2.0 | 3.0 | 3.0 | 1.8 | na | 1.4 | 17.0 | na | 1.7 |  |
|  | **Rural household** | 7.6 | 22.3 | 6.3 | na | na | 1.5 | 3.3 | 8.0 | 1.5 | 18.3 | na | 1.0 |  |
| ^1^ Animal ownership was a criterion for enrollment; several households reported ownership of multiple animal types | | | | | | | | | | | | | | |

| **Table S2:** Total household samples collected for MST testing. | |
| --- | --- |
| Sample Type | *n* |
| Adult hands | 59 |
| Cellphone | 30 |
| Child hands | 31 |
| Dish drying surface | 10 |
| Domestic water | 48 |
| Doorknob | 59 |
| Drinking water | 55 |
| Floor | 59 |
| Food | 57 |
| Food- prep surface | 57 |
| House keys | 19 |
| Soil | 54 |
| TV remote control | 16 |
| Toy | 31 |

| **Table S3:** Summary of sample characteristics. | | | |
| --- | --- | --- | --- |
| **Sample Type** | **Household Environmental Practice** | **Response** | **n (%)** |
| Adult hands | Adult clean hands | No | 41 (69.5%) |
|  |  | Yes | 18 (30.5%) |
|  | Adult clean nails | No | 30 (50.8%) |
|  |  | Yes | 29 (49.2%) |
|  | Adult last washed | Less than one hour ago | 28 (47.5%) |
|  |  | More than two hours ago | 9 (15.3%) |
|  |  | One hour ago | 8 (13.6%) |
|  |  | Two hours ago | 14 (23.7%) |
| Child hands | Child clean hands | No | 20 (64.5%) |
|  |  | Yes | 11 (35.5%) |
|  | Child clean nails | No | 11 (35.5%) |
|  |  | Yes | 20 (64.5%) |
|  | Child last washed | Less than one hour ago | 6 (19.4%) |
|  |  | More than two hours ago | 7 (22.6%) |
|  |  | One hour ago | 11 (35.5%) |
|  |  | Two hours ago | 7 (22.6%) |
| Domestic water | Animals access domestic water | Missing data | 3 (6.2%) |
|  |  | No | 42 (87.5%) |
|  |  | Yes | 3 (6.2%) |
|  | Child access to domestic water | Missing data | 26 (54.2%) |
|  |  | No | 14 (29.2%) |
|  |  | Yes | 8 (16.7%) |
|  | Domestic water container type | Missing data | 12 (25%) |
|  |  | Not covered | 22 (45.8%) |
|  |  | Poorly covered | 3 (6.2%) |
|  |  | Well covered | 11 (22.9%) |
|  | Domestic water stored | Missing data | 3 (6.2%) |
|  |  | No | 9 (18.8%) |
|  |  | Yes | 36 (75%) |
| Doorknob | Door location | Entrance door | 53 (89.8%) |
|  |  | Kitchen door | 3 (5.1%) |
|  |  | Patio exit door | 3 (5.1%) |
| Drinking water | Drinking water container type | Missing data | 3 (5.5%) |
|  |  | Not covered | 9 (16.4%) |
|  |  | Poorly covered | 14 (25.5%) |
|  |  | Well covered | 29 (52.7%) |
|  | Drinking water source | Bottled water | 32 (58.2%) |
|  |  | Piped water connection | 10 (18.2%) |
|  |  | Protected well | 1 (1.8%) |
|  |  | Rainwater | 11 (20%) |
|  |  | Unprotected well | 1 (1.8%) |
|  | Drinking water stored | No | 3 (5.5%) |
|  |  | Yes | 52 (94.5%) |
|  | Drinking water treated | Boil | 3 (5.5%) |
|  |  | Chlorine | 1 (1.8%) |
|  |  | Filter | 2 (3.6%) |
|  |  | Larvicide | 2 (3.6%) |
|  |  | None | 47 (85.5%) |
| Floor | Floor cleaned in the past 24h | No | 50 (84.7%) |
|  |  | Yes | 9 (15.3%) |
|  | Floor type | Carpet | 1 (1.7%) |
|  |  | Cement | 22 (37.3%) |
|  |  | Ceramic tiles | 16 (27.1%) |
|  |  | Other | 1 (1.7%) |
|  |  | Vinyl/asphalt | 4 (6.8%) |
|  |  | Wooden boards | 15 (25.4%) |
| Food | Food container | Missing data | 7 (12.3%) |
|  |  | Not covered | 21 (36.8%) |
|  |  | Poorly covered | 15 (26.3%) |
|  |  | Well covered | 14 (24.6%) |
|  | Food temperature | Missing data | 7 (12.3%) |
|  |  | Refrigerated | 9 (15.8%) |
|  |  | Room temperature | 41 (71.9%) |
|  | Food type | Missing data | 4 (7%) |
|  |  | Colada | 3 (5.3%) |
|  |  | Cooked rice | 47 (82.5%) |
|  |  | Green plantain | 2 (3.5%) |
|  |  | Other food | 1 (1.8%) |
| Food-prep surface | Food-prep surface last cleaned | No | 39 (68.4%) |
|  |  | Yes | 18 (31.6%) |
|  | Food-prep surface material | Cement | 6 (10.5%) |
|  |  | Ceramic tiles | 24 (42.1%) |
|  |  | Other | 3 (5.3%) |
|  |  | Plastic | 9 (15.8%) |
|  |  | Stainless steel | 1 (1.8%) |
|  |  | Wood | 14 (24.6%) |
| Soil | Soil moisture | Dry | 16 (29.6%) |
|  |  | Moist | 32 (59.3%) |
|  |  | Very wet | 6 (11.1%) |
|  | Visible animal feces | No | 46 (85.2%) |
|  |  | Yes | 8 (14.8%) |
|  | Visible human feces | No | 31 (57.4%) |
|  |  | Yes | 23 (42.6%) |
| Toy | Toy dirty/clean | No | 29 (93.5%) |
|  |  | Yes | 2 (6.5%) |
|  | Toy material | Cotton | 1 (3.2%) |
|  |  | Plastic | 29 (93.5%) |
|  |  | Wood | 1 (3.2%) |

| **TABLE S4:** Performance of MST qPCR assays in the validation study. | | | | | |
| --- | --- | --- | --- | --- | --- |
| **Assay** | **Slope** | **Intercept** | **R^2^** | **Amplification Efficiency** | **LOD (GC/rxn)** |
| GenBac3 | -3.514 ± 0.023 | 40.635 ± 0.710 | 0.993 ± 0.009 | 0.926 ± 0.008 | 10 |
| HF183 | -3.414 ± 0.096 | 39.024 ± 0.834 | 0.985 ± 0.011 | 0.965 ± 0.037 | 5 |
| HumM2 | -3.432 ± 0.117 | 40.104 ± 0.423 | 0.992 ± 0.008 | 0.959 ± 0.044 | 5 |
| DG37 | -3.485 ± 0.086 | 39.841 ± 1.035 | 0.992 ± 0.008 | 0.939 ± 0.028 | 5 |
| GFD | -3.364 ± 0.141 | 40.897 ± 0.998 | 0.989 ± 0.008 | 0.986 ± 0.059 | 5 |
| AV4143 | -3.557 ± 0.106 | 39.445 ± 0.995 | 0.987 ± 0.009 | 0.925 ± 0.022 | 5 |
| Rum2Bac | -3.35 ± 0.121 | 40.835 ± 0.926 | 0.974 ± 0.019 | 0.991 ± 0.052 | 5 |
| Pig2Bac | -3.368 ± 0.121 | 41.236 ± 1.882 | 0.989 ± 0.007 | 0.983 ± 0.050 | 5 |

| **Table S5:** Performance of MST qPCR assays in the household sample analyses. | | | | | |
| --- | --- | --- | --- | --- | --- |
| **Assay** | **Slope** | **Intercept** | **R²** | **Amplification Efficiency** | **LOD (GC/rxn)** |
| GenBac3 | -3.397 ± 0.070 | 39.474 ± 1.053 | 0.994 ± 0.008 | 0.970 ± 0.027 | 10 |
| HF183 | -3.398 ± 0.075 | 36.241 ± 0.681 | 0.995 ± 0.003 | 0.970 ± 0.029 | 5 |
| DG37 | -3.347 ± 0.117 | 37.444 ± 0.933 | 0.992 ± 0.006 | 0.992 ± 0.048 | 5 |
| GFD | -3.364 ± 0.086 | 37.078 ± 1.009 | 0.994 ± 0.005 | 0.984 ± 0.034 | 5 |
| Rum2Bac | -3.397 ± 0.070 | 39.474 ± 1.053 | 0.994 ± 0.008 | 0.970 ± 0.027 | 5 |
| Pig2Bac | -3.398 ± 0.075 | 36.241 ± 0.681 | 0.995 ± 0.003 | 0.970 ± 0.029 | 5 |

| **Table S6:** Adjusted *p*-values for pairwise post hoc Fisher’s tests following a global Fisher’s test (*p*<0.001) for differences in GenBac3 prevalence between sample types. Values were adjusted for multiple comparisons using the Benjamini-Hochberg method. Significance at *p*<0.05 is indicated with bold typeface. | | | | | | | | | | | | | |
| --- | --- | --- | --- | --- | --- | --- | --- | --- | --- | --- | --- | --- | --- |
|  | Adult hands | Cellphone | Child hands | Dish drying surface | Domestic water | Doorknob | Drinking water | Floor | Food | Food- prep surface | House keys | Soil | TV remote control |
| Cellphone | 0.487 |  |  |  |  |  |  |  |  |  |  |  |  |
| Child hands | 1 | 0.639 |  |  |  |  |  |  |  |  |  |  |  |
| Dish drying surface | 0.259 | 0.592 | 0.396 |  |  |  |  |  |  |  |  |  |  |
| Domestic water | **0**.**003** | 0.258 | **0.041** | 1 |  |  |  |  |  |  |  |  |  |
| Doorknob | 1 | 1 | 1 | 0.413 | 0.025 |  |  |  |  |  |  |  |  |
| Drinking water | **<0.001** | **0.011** | **0.001** | 0.413 | 0.289 | **<0.001** |  |  |  |  |  |  |  |
| Floor | 0.057 | 0.565 | 0.177 | 1 | 0.542 | 0.216 | **0.036** |  |  |  |  |  |  |
| Food | **<0.001** | **<0.001** | **<0.001** | **<0.001** | **<0.001** | **<0.001** | **<0.001** | **<0.001** |  |  |  |  |  |
| Food- prep surface | 0.396 | 1 | 0.684 | 0.542 | 0.083 | 0.746 | **<0.001** | 0.413 | **<0.001** |  |  |  |  |
| House keys | **<0.001** | **0.01** | **0.002** | 0.338 | 0.202 | **<0.001** | 0.714 | **0.029** | **0.004** | **0.002** |  |  |  |
| Soil | **<0.001** | **<0.001** | **<0.001** | **<0.001** | **<0.001** | **<0.001** | **<0.001** | **<0.001** | 0.784 | **<0.001** | **0.014** |  |  |
| TV remote control | 1 | 1 | 1 | 0.542 | 0.317 | 1 | **0.034** | 0.486 | **<0.001** | 1 | **0.023** | **<0.001** |  |
| Toy | 0.216 | 1 | 0.639 | 1 | 0.448 | 0.413 | **0.032** | 0.839 | **<0.001** | 0.746 | **0.04** | **<0.001** | 0.684 |

| **Table S7:**Pairwise post hoc Dunn’s test results for differences in GenBac3 concentrations between sample types; *p* adjusted values <0.05 were considered statistically significant and are indicated by bold typeface. | | | | |
| --- | --- | --- | --- | --- |
| **Group 1** | **Group 2** | **Dunn statistic** | ***p*** | ***p* adjusted** |
| Adult hands | Drinking water | -12.13 | 6.91E-34 | **4.56E-32** |
| Child hands | Drinking water | -10.98 | 4.80E-28 | **1.58E-26** |
| Adult hands | Food- prep surface | -9.31 | 1.30E-20 | **2.85E-19** |
| Child hands | Food- prep surface | -8.60 | 7.67E-18 | **1.27E-16** |
| Adult hands | Floor | -8.27 | 1.33E-16 | **1.76E-15** |
| Child hands | Floor | -7.73 | 1.10E-14 | **1.21E-13** |
| Adult hands | House keys | -7.62 | 2.49E-14 | **2.35E-13** |
| Child hands | House keys | -7.56 | 4.10E-14 | **3.38E-13** |
| Adult hands | Domestic water | -7.53 | 5.03E-14 | **3.69E-13** |
| Child hands | Domestic water | -7.19 | 6.43E-13 | **4.24E-12** |
| Drinking water | Toy | 7.14 | 9.50E-13 | **5.70E-12** |
| Doorknob | Drinking water | -6.43 | 1.24E-10 | **6.83E-10** |
| Child hands | Dish drying surface | -6.26 | 3.84E-10 | **1.95E-09** |
| Adult hands | Cellphone | -6.13 | 8.84E-10 | **4.17E-09** |
| Cellphone | Child hands | 6.11 | 9.74E-10 | **4.29E-09** |
| Adult hands | Dish drying surface | -6.10 | 1.07E-09 | **4.43E-09** |
| Adult hands | Doorknob | -5.80 | 6.50E-09 | **2.52E-08** |
| Child hands | Doorknob | -5.68 | 1.35E-08 | **4.95E-08** |
| Food- prep surface | Toy | 4.74 | 2.16E-06 | **7.50E-06** |
| House keys | Toy | 4.60 | 4.30E-06 | **1.42E-05** |
| Drinking water | TV remote control | 4.24 | 2.27E-05 | **7.14E-05** |
| Child hands | TV remote control | -4.10 | 4.11E-05 | **1.23E-04** |
| Domestic water | Drinking water | -4.04 | 5.40E-05 | **1.55E-04** |
| Drinking water | Floor | 4.01 | 6.06E-05 | **1.67E-04** |
| Cellphone | Drinking water | -3.97 | 7.32E-05 | **1.93E-04** |
| Dish drying surface | Toy | 3.89 | 1.01E-04 | **2.57E-04** |
| Floor | Toy | 3.84 | 1.24E-04 | **3.04E-04** |
| Adult hands | TV remote control | -3.80 | 1.45E-04 | **3.41E-04** |
| Doorknob | House keys | -3.57 | 3.54E-04 | **8.07E-04** |
| Doorknob | Food- prep surface | -3.56 | 3.77E-04 | **8.30E-04** |
| Domestic water | Toy | 3.46 | 5.36E-04 | **1.14E-03** |
| Child hands | Toy | -3.40 | 6.81E-04 | **1.41E-03** |
| Adult hands | Toy | -3.03 | 2.47E-03 | **4.94E-03** |
| Dish drying surface | Doorknob | 2.97 | 2.94E-03 | **0.01** |
| Drinking water | Food- prep surface | 2.89 | 3.89E-03 | **0.01** |
| House keys | TV remote control | 2.77 | 0.01 | **0.01** |
| Cellphone | Toy | 2.74 | 0.01 | **0.01** |
| Dish drying surface | TV remote control | 2.52 | 0.01 | **0.02** |
| Doorknob | Floor | -2.47 | 0.01 | **0.02** |
| Food- prep surface | TV remote control | 2.32 | 0.02 | **0.03** |
| Cellphone | House keys | -2.17 | 0.03 | 0.05 |
| Domestic water | Doorknob | 2.07 | 0.04 | 0.06 |
| Domestic water | House keys | -1.98 | 0.05 | 0.07 |
| Cellphone | Dish drying surface | -1.95 | 0.05 | 0.08 |
| Floor | House keys | -1.85 | 0.06 | 0.09 |
| Doorknob | Toy | 1.79 | 0.07 | 0.11 |
| Dish drying surface | Domestic water | 1.76 | 0.08 | 0.11 |
| Dish drying surface | Floor | 1.65 | 0.10 | 0.14 |
| Floor | TV remote control | 1.60 | 0.11 | 0.15 |
| Cellphone | Food- prep surface | -1.57 | 0.12 | 0.15 |
| Domestic water | TV remote control | 1.39 | 0.17 | 0.21 |
| Cellphone | Doorknob | 1.36 | 0.17 | 0.22 |
| Toy | TV remote control | -1.30 | 0.19 | 0.24 |
| Domestic water | Food- prep surface | -1.30 | 0.19 | 0.24 |
| Floor | Food- prep surface | -1.11 | 0.27 | 0.32 |
| Food- prep surface | House keys | -1.06 | 0.29 | 0.34 |
| Dish drying surface | Food- prep surface | 1.04 | 0.30 | 0.35 |
| Drinking water | House keys | 0.99 | 0.32 | 0.37 |
| Cellphone | TV remote control | 0.98 | 0.33 | 0.37 |
| Adult hands | Child hands | 0.86 | 0.39 | 0.43 |
| Cellphone | Floor | -0.66 | 0.51 | 0.55 |
| Dish drying surface | Drinking water | -0.55 | 0.58 | 0.62 |
| Cellphone | Domestic water | -0.42 | 0.67 | 0.71 |
| Domestic water | Floor | -0.26 | 0.80 | 0.82 |
| Dish drying surface | House keys | 0.19 | 0.85 | 0.86 |
| Doorknob | TV remote control | -0.01 | 0.99 | 0.99 |

| **Table S8:** Adjusted *p*-values for pairwise post hoc Fisher’s tests following a global Fisher’s test (*p*<0.001) for differences in HF183 prevalence between sample types. Values were adjusted for multiple comparisons using the Benjamini-Hochberg method. Significance at *p*<0.05 is indicated with bold typeface. | | | | | | | | | | | | | |
| --- | --- | --- | --- | --- | --- | --- | --- | --- | --- | --- | --- | --- | --- |
|  | Adult hands | Cellphone | Child hands | Dish drying surface | Domestic water | Doorknob | Drinking water | Floor | Food | Food- prep surface | House keys | Soil | TV remote control |
| Cellphone | **<0.001** |  |  |  |  |  |  |  |  |  |  |  |  |
| Child hands | 1 | **<0.001** |  |  |  |  |  |  |  |  |  |  |  |
| Dish drying surface | **0.006** | 1 | **0.024** |  |  |  |  |  |  |  |  |  |  |
| Domestic water | **<0.001** | 0.565 | **0.002** | 0.757 |  |  |  |  |  |  |  |  |  |
| Doorknob | **<0.001** | 0.573 | **<0.001** | 0.757 | 1 |  |  |  |  |  |  |  |  |
| Drinking water | **<0.001** | 0.516 | **<0.001** | 1 | 0.054 | 0.062 |  |  |  |  |  |  |  |
| Floor | **0.045** | **0.016** | 0.189 | 0.109 | 0.063 | 0.054 | **<0.001** |  |  |  |  |  |  |
| Food | **<0.001** | 0.514 | **<0.001** | 1 | 0.054 | 0.062 | 1 | **<0.001** |  |  |  |  |  |
| Food- prep surface | **<0.001** | 0.573 | **<0.001** | 0.757 | 1 | 1 | 0.062 | 0.054 | 0.062 |  |  |  |  |
| House keys | **<0.001** | 1 | **0.001** | 1 | 0.496 | 0.501 | 1 | **0.027** | 1 | 0.501 |  |  |  |
| Soil | **<0.001** | 0.516 | **<0.001** | 1 | 0.055 | 0.062 | 1 | **<0.001** | 1 | 0.062 | 1 |  |  |
| TV remote control | **0.016** | 0.445 | 0.062 | 0.69 | 1 | 0.855 | 0.098 | 0.501 | 0.094 | 1 | 0.34 | 0.098 |  |
| Toy | **0.025** | 0.102 | 0.115 | 0.288 | 0.34 | 0.225 | **0.002** | 0.795 | **0.002** | 0.342 | 0.075 | **0.002** | 0.869 |

| **Table S9:** Adjusted *p*-values for pairwise post hoc Fisher’s tests following a global Fisher’s test (*p*<0.001) for differences in DG37 prevalence between sample types. Values were adjusted for multiple comparisons using the Benjamini-Hochberg method. There were no significant results at *p*<0.05 for the pairwise tests. | | | | | | | | | | | | | |
| --- | --- | --- | --- | --- | --- | --- | --- | --- | --- | --- | --- | --- | --- |
|  | Adult hands | Cellphone | Child hands | Dish drying surface | Domestic water | Doorknob | Drinking water | Floor | Food | Food- prep surface | House keys | Soil | TV remote control |
| Cellphone | 1 |  |  |  |  |  |  |  |  |  |  |  |  |
| Child hands | 0.929 | 0.741 |  |  |  |  |  |  |  |  |  |  |  |
| Dish drying surface | 1 | 1 | 0.98 |  |  |  |  |  |  |  |  |  |  |
| Domestic water | 0.603 | 1 | 0.086 | 1 |  |  |  |  |  |  |  |  |  |
| Doorknob | 0.98 | 1 | 0.144 | 1 | 1 |  |  |  |  |  |  |  |  |
| Drinking water | 0.603 | 0.98 | 0.063 | 1 | 1 | 1 |  |  |  |  |  |  |  |
| Floor | 0.87 | 0.64 | 1 | 0.98 | 0.063 | 0.144 | 0.063 |  |  |  |  |  |  |
| Food | 0.603 | 0.98 | 0.063 | 1 | 1 | 1 | 1 | 0.063 |  |  |  |  |  |
| Food- prep surface | 0.603 | 0.98 | 0.063 | 1 | 1 | 1 | 1 | 0.063 | 1 |  |  |  |  |
| House keys | 1 | 1 | 0.64 | 1 | 1 | 1 | 1 | 0.603 | 1 | 1 |  |  |  |
| Soil | 0.98 | 1 | 0.172 | 1 | 1 | 1 | 1 | 0.144 | 1 | 1 | 1 |  |  |
| TV remote control | 1 | 1 | 0.64 | 1 | 1 | 1 | 1 | 0.741 | 1 | 1 | 1 | 1 |  |
| Toy | 0.98 | 1 | 0.341 | 1 | 1 | 1 | 1 | 0.174 | 1 | 1 | 1 | 1 | 1 |

| **Table S10:** Adjusted *p*-values for pairwise post hoc Fisher’s tests following a global Fisher’s test (*p*<0.001) for differences in GFD prevalence between sample types. Values were adjusted for multiple comparisons using the Benjamini-Hochberg method. Significance at *p*<0.05 is indicated with bold typeface. | | | | | | | | | | | | | |
| --- | --- | --- | --- | --- | --- | --- | --- | --- | --- | --- | --- | --- | --- |
|  | Adult hands | Cellphone | Child hands | Dish drying surface | Domestic water | Doorknob | Drinking water | Floor | Food | Food- prep surface | House keys | Soil | TV remote control |
| Cellphone | 1 |  |  |  |  |  |  |  |  |  |  |  |  |
| Child hands | 1 | 1 |  |  |  |  |  |  |  |  |  |  |  |
| Dish drying surface | 1 | 1 | 1 |  |  |  |  |  |  |  |  |  |  |
| Domestic water | 1 | 1 | 0.858 | 1 |  |  |  |  |  |  |  |  |  |
| Doorknob | 1 | 1 | 1 | 1 | 1 |  |  |  |  |  |  |  |  |
| Drinking water | 1 | 1 | 1 | 1 | 1 | 1 |  |  |  |  |  |  |  |
| Floor | **0.039** | **0.021** | 0.341 | 0.724 | **0.003** | **0.016** | **0.004** |  |  |  |  |  |  |
| Food | 1 | 1 | 0.79 | 1 | 1 | 1 | 1 | **0.001** |  |  |  |  |  |
| Food- prep surface | 1 | 1 | 1 | 1 | 1 | 1 | 1 | **0.004** | 1 |  |  |  |  |
| House keys | 1 | 1 | 1 | 1 | 1 | 1 | 1 | 0.147 | 1 | 1 |  |  |  |
| Soil | 1 | 1 | 0.79 | 1 | 1 | 1 | 1 | **0.001** | 1 | 1 | 1 |  |  |
| TV remote control | 1 | 1 | 1 | 1 | 1 | 1 | 1 | 0.255 | 1 | 1 | 1 | 1 |  |
| Toy | 1 | 1 | 1 | 1 | 1 | 1 | 1 | 0.**021** | 1 | 1 | 1 | 1 | 1 |

| **Table S11:** Association between HF183 detection on adult hands and other household sample types. Risk ratios and 95% confidence intervals (Cis) were estimated using Poisson regression with generalized estimating equations (GEE) to account for clustering at the household level. Each comparison represents a separate model restricted to households with paired samples. | | | | | |
| --- | --- | --- | --- | --- | --- |
| Comparison | Risk Ratio | Lower 95% CI | Upper 95% CI | *p* | *n* |
| Child hands | 4.27 | 1.49 | 12.2 | 0.00681 | 31 |
| Floor | 6.77 | 1.7 | 27.04 | 0.00675 | 59 |
| Food- prep surface | 4.5 | 0.56 | 36.13 | 0.157 | 57 |
